# Development and preliminary evaluation of the participation in life activities scale for children and adolescents with asthma: an instrument development study

**DOI:** 10.1186/1477-7525-6-37

**Published:** 2008-05-28

**Authors:** Eileen K Kintner

**Affiliations:** 1 Michigan State University College of Nursing, East Lansing, MI, USA

## Abstract

**Background:**

Being able to do things other kids do is the desire of school-age children and adolescents with asthma. In a phenomenology study, adolescents identified participation in life activities as the outcome variable and primary motivator for behavioral changes in coming to accept asthma as a chronic condition. In preparation for testing an acceptance model for older school-age children and early adolescents diagnosed with asthma, the Participation in Life Activities Scale was developed. The purposes of this paper are to describe development, and report on face and content validity of the scale designed to measure one aspect of quality of life defined as level of unrestricted involvement in chosen pursuits.

**Methods:**

Items generated for the instrument evolved from statements and themes extracted from qualitative interviews. Face and content validity were evaluated by eight lay reviewers and 10 expert reviewers. Rate of accurate completion was computed using a convenience, cross-section sample consisting of 313 children and adolescents with asthma, ages 9–15 years, drawn from three studies. Preliminary cross-group comparisons of scores were assessed using t-tests and analysis of variance.

**Results:**

Face and content validity were determined to be highly acceptable and relevant, respectively. Completion rate across all three studies was 97%. Although cross-group comparisons revealed no significant differences in overall participation scores based on age, race or residence groupings (*p *> .05), significant difference were indicated between males and females (*p *= .02), as well as the highest and lowest socioeconomic groups (*p *= .002).

**Conclusion:**

Assessing content validity was the first step in evaluating properties of this newly developed instrument. Once face and content validity were established, psychometric evaluation related to internal consistency reliability and construct validity using factor analysis procedures was begun. Results will be reported elsewhere.

## Background

Asthma is the leading chronic condition of childhood and leading cause of disability in this group [1]. Nine million (7–17%) children in the United States less than age 18 years have been diagnosed with asthma at some point in their lives and more than 4 million (6%) children have experienced an acute episode in the last 12 months [2]. Exposure to symptom-stimulating situations, often restricts children with asthma from participating in everyday activities such as laughing with friends, swimming in chlorinated pools, riding horses, playing with pets, going to camp, eating certain foods, being indoor or outdoor, exercising, and sleeping [3-9]. School absences in students with asthma are 3 times higher than those of students without asthma [10]. Being able to do things other kids do is the desire of children and adolescents with asthma [11,12].

In 1994 a qualitative study was conducted to identify the essential structure of the adolescent process of coming to accept asthma as a chronic condition [12]. One outcome of the shared lived experience was the Acceptance of Asthma Model [12], a process model with the major positive outcome being full participation in life activities. This outcome variable is defined as unrestricted involvement in chosen pursuits, such as clubs, sports, interests, and hobbies [13]. In preparation for theory testing, a measure consistent with the definition was developed, the Participation in Life Activities Scale (PLA) [13-15].

### Purpose

The purposes of this paper are to describe development, and report on face and content validity of the Participation in Life Activities Scale (PLA) for children and adolescents with asthma. Development considers domain identification, item generation, and instrument formation [16]. Face-valid measures require evaluation by representatives of the target population [17]. Content validity is the determination of the item relevance by experts using a judgment or quantification process [16]. Establishing face and content validity are the first steps in evaluating properties of newly developed instruments. Once face and content validity are established, psychometric testing is possible.

### Theoretical framework

#### Foundational assumptions

The PLA was developed, as an outcome measure for child and adolescent acceptance of asthma, to measure one aspect of quality of life believed to influence one's overall quality of life. Adolescents with asthma identified level of participation in activities as their prime motivator for behavioral changes in coming to accept asthma as a chronic condition requiring ongoing monitoring and management [12]. Based on preliminary work, the following assumptions were identified as important considerations in development of the scale:

1. Level of participation in self-selected activities offers a measure of one aspect of quality of life.

2. Severity of illness restricts participation in favorite activities thus impacting one's overall quality of life.

3. Level of symptom control through use of proper medical treatments and effective management techniques allows for full participation in life activities.

#### The Lifespan Development perspective

[18-21] and Acceptance of Asthma Model [12-15] were used to guide development of the PLA. Lifespan Development is an orientation, providing conceptual and methodological framing for the study of human development and change processes. Principles of Lifespan Development hold that individuals are producers of their own development with the assumption that developmental change in a structure proceeds toward increasing complexity, differentiation, and specialization; while increasing in hierarchical integration and organization [18-21]. The potential for development extends throughout life, across various dimensions, in multiple directions, and on many different levels, often independent of growth. Non-normative events, such as experiences with asthma, are major contributing factors of development. Interventions are moderated by a wide range of factors and vary across individuals. This perspective highlights the importance of having participants with asthma select their activities and allowing the activities to change as children grow and develop from age 8–18 years.

#### The Acceptance of Asthma Model

describes how children come to terms with their chronic condition [12-15]. The process is hypothesized to begin with an awareness of symptoms that leads the family to seek assistance from healthcare professionals who acknowledge the symptoms through a diagnosis and prescription for treatment. Asthma specific episode management, risk reduction/preventative, and health promotion behaviors are tried to manage the condition. To gain knowledge, information about the diagnosis is sought. Based on the effectiveness of health behaviors implemented, a period of resignation ensues as children are challenged to understand the impact of limitations. As they develop reasoning abilities, children explore options and choices, and cause and effect relationships. Reasoning leads to drawing conclusions about the condition that resolves turmoil caused by negative emotions. They form beliefs for accepting the condition that ushers in the potential for participation in life activities. Disease and individual characteristics, and environmental factors are believed to influence children as they move though the process. Table 1 contains the indicators that distinguish participation in life activities from other concepts as well as presents definitions, guiding principles, and referent statements for the indicators based on findings from the qualitative study.

**Table 1:** Concept and Indicator Definitions, Guiding Principles, and Qualitative Study Referents

**Concept & Indicators**	**Definition**	**Guiding Principles**	**Qualitative Study Referent [12]**
Participation in Life Activities	A child's or adolescent's unrestricted involvement in chosen pursuits, such as sports, clubs, interests, and hobbies.	Subjects self-select up to five or more of their most favorite or desirable activities.	Whereas some participants were not interested in sports, others competed at state, national, and international levels.
		• Activities are allowed to change over time as children grow and develop.	* *I didn't grow up with sports and wasn't around sports so I am not as interested in sports. I'm student director of our youth group. My asthma is no big deal. I only take medication as needed*.
		• The activities are not as important as the level of restriction from participation believed to motivate changes in self management.	* *Everybody needs to succeed at something: chess, academics, art or sports. Success is what makes you. I'm good at swimming*.
**Indicators**			
1. Planning for Participation	The amount of thinking about the condition required before engaging in desired activities.	With proper treatment and management, children with asthma should be able to participate in the same activities and at the same level as children without asthma.	Participation sometimes required planning.
		• Children may sometimes need to consider their asthma when planning for activities.	* *Now that I'm going to be starting cheerleading, I have to start taking asthma medication every day. I will also need to carry my inhaler with me*.
			* *When leaving to play basketball, my friends ask me if I have my inhaler because they don't want to have to come back if I have breathing problems*.
2. Interference with Participation	The amount of temporary disruption with engaging in desired activities due to the condition.	• Children should rarely allow asthma to interfere with or disrupt participation.	Participants shared thoughts and feelings of times asthma interfered with participation.
			* *I went on a hayride with my friends and started having asthma problems around the campfire that evening*.
			* *I hate having to sit out and watch because of my asthma*.
3. Prevention from Participation	The amount of complete limitation from engaging in desired activities due to the condition.	• Children should almost never allow asthma to prevent participation.	Where some participants were prevented from caring for pets, others followed medical treatment plans and used management techniques so that participation was possible.
			* *I want to have a pet to care for, but can't because of my asthma*.
			* *Living on a farm, I have to take my medication everyday so I can care for my horse and play with the dogs*.

### Review of quality of life measures and domains of activity limitations

The newly developed PLA offers a unique qualitatively-derived, first person, emic, perspective and theory-based method for measuring what children and adolescents identify as their primary motivator for behavioral change in coming to accept asthma as a chronic condition, specifically unrestricted or rather full participation in self-selected life activities. Although a few global quality of life instruments contain items that address physical activities and limitations, conceptual and operational definitions for the PLA provide transparency in measurement of participation in life activities. In addition, indicators based on statements of children and adolescents with asthma distinguish this concept from concepts measured by other quality of life instruments.

#### Life activities measure

The Life Activities Questionnaire for Childhood Asthma (LAQ) [22] was initially considered for measuring the concept. The 52-item, 5-point Likert-type, instrument was designed to measure the degree to which children believed they were restricted from engaging in activities in the past week. The instrument lists activities grouped under categories of physical, work, outdoor, emotional, home care, eating and drinking, and miscellaneous. A content review of the LAQ by this author resulted in questions about completion rates, appropriateness, usefulness, and applicability for children. The instrument was long and for children not interested in participating in strenuous activities, the list of athletic activities could be disconcerting. Because most children are not employed, the work-related items were inappropriate. Some outdoor activities (e.g. mowing the grass, raking leaves, shovelling snow, and cutting wood) and home care items (e.g. dusting, cleaning the basement or garage, and scrubbing floors) presented more as chores than activities of interest that would motivate the use self-management behaviors. In addition, many activities appeared to be regionally specific to the Midwest and not as appropriate to other areas of the United States, such as the desert Southwest. Consequently, a new instrument needed to be developed.

Concurrent to testing of the PLA, and because of limitations of the LAQ other instruments [23-25] were being developed for children with asthma to measure more global constructs of quality of life. Items contained in some of the instruments addressed domains of activity limitation.

#### The Pediatric Asthma Quality of Life Questionnaire

(PAQLQ) is a 23-item, 7-point scale, designed to measure quality of life in three domains: activity limitation, symptoms, and emotional function [24]. The activity limitation domain contains five items, three of which are individualized. Children are asked to identify three activities that were limited due to their asthma in the recent past, important to the child, and performed frequently. The activities are retained for future use. Two additional items ask about how often participants could not keep up with others and how much they were bothered by asthma while participating in activities during the past week.

Developers of the PAQLQ evaluated content validity through peer and expert review. Although the PAQLQ has been translated into more than 30 languages and is used widely throughout the world [26], the structure does not lend itself to psychometric testing. Using a sample of 52 children and adolescents with asthma, ages 7–17 years, clinimetrics based on t-tests and correlations were used to examine evaluative and discriminative capabilities [24]. In patients whose health state was deemed unchanged, the scale had an acceptable stability coefficient (ICC = .84). In patients whose health state was believed to have changed, the scale was deemed responsive (*p *< 0.0001). Weak to moderate correlations were reported with severity measures.

Although the PAQLQ has been deemed to be of some clinical value over limited periods of time, using the instrument to test theory or evaluate the efficacy of theory-based interventions could be problematic due to the varied presentations of structure, format, and content as well as choice of items and response options. Life activities change with seasons and overtime as children grow and develop. Selecting three activities that were limiting in the recent past for future use at 6–12 weeks, 18–24 months or 3–4 years is problematic. For example, with only sport activities considered, hockey or skating might be the focus during winter months that turn to volleyball in summer or soccer/football in fall. Comparing running outside during winter with cold air as a stimulus to spring with pollen, summer with ragweed or fall with mold induces measurement error. Students enrolled in fifth grade might be members of a baseball team, whereas by seventh grade be disinterested in baseball and involved in competitive swimming. Variability induced by placing weight on the specific activity is of concern when evaluating progression of condition and effectiveness of treatments or interventions over time. Activities that might have been limiting last week may not possess motivating effects into the future.

#### The Pediatric Quality of Life Inventory™ Generic Core Scales and Asthma Module

(PedsQL™) is a 28-item, 5-point Likert-type, scale designed to measure health-related quality of life in children, ages 2–18 years, based on frequency of problems with physical symptoms, treatment, worry, and communication [25]. Although the instrument has demonstrated internal consistency, stability and ability to measure change, and construct validity; only two items contained in the "problems with physical symptoms" section address activities. The items ask: How often was it hard to play with pets and to play outside?

#### The Adolescent Asthma Quality of Life Questionnaire

(AAQOL) is a 32-item scale containing six domains: symptoms, medication, physical activities, emotion, social interaction, and positive effects [23]. This was designed to measure how frequently events happened and how important the events are to the participant. Six physical activity items ask about frequency and importance of symptoms associated with running, difficulty with long distance sports, avoiding things that worsen symptoms, restriction in general activities, school absenteeism, and difficulty walking upstairs. Using a sample of 111 adolescents, ages 12–17 years, Cronbach's alpha correlation coefficient for internal consistency was .85. Using 20 stable participants, test-retest reliability was good for all domains (ICC = .76–.85). Spearman rank correlations revealed weak to moderate associations with health outcomes and asthma severity.

Although the LAQ [22] and PAQLQ [24], and to some degree PedsQL™ [25] are considered to measure domains of physical limitations, the scales were deemed inadequate or inappropriate to measure the concept as defined by participants in the qualitative study who identified participation in self-selected activities as their prime motivator for effective self-management. The AAQOL physical activity subscale [23] could be used as a global measure of limitation to evaluate convergent validity of the PLA.

## Methods

### Development of the PLA

The PLA scale is a 15-question, 3-indicator scale designed to measure level of unrestricted involvement in chosen life activities. The questionnaire completed by the child is titled "*My Favorite Things to Do*." [see Additional file 1] Subjects are asked to list their favorite activities then answer three questions about each of them. The activities are not as important as their motivating influences. The three questions are reflective of indicators that evolved from statements and themes extracted from qualitative interviews. The scale was written at a fourth grade comprehension level.

#### Activities

A list of activities categorized under clubs, crafts, and sports is provided. Subjects may choose from the list or select other activities. Because participation in activities was the prime motivator for behavioral change by adolescents who were accepting of their asthma, having subjects select their own activities is imperative. When children are not vested in activities, then little will motivate the non-normative behaviorial changes necessary for managing a chronic condition. Numbers and types of activities must also be allowed to vary as children increase in complexity, differentiation, and specialization; while increasing in hierarchical integration and organization.

#### Indicators

Three questions address each activity asking whether or not subjects need to think about their asthma when planning for participation, and whether or not asthma interferes with or prevents participation. Directions include examples of thought processes necessary for answering the questions. The activity or classification of activity referred to by the question is not as important as whether or not planning is required and/or participation is disrupted or limited. The three indicators measured by the activity-specific questions are cited below:

1. How much thinking about asthma is required when planning for participation in your favorite activities?

2. How much does asthma interfere with or disrupt participation in your favorite activities?

3. How much does asthma completely prevent participation in your favorite activities?

#### Scoring

**Figure 1:**
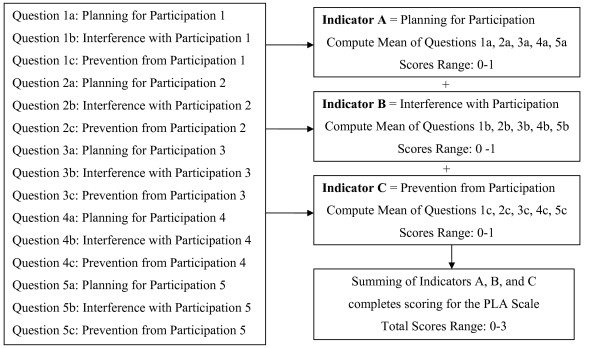
Scoring of the Participation in Life Activities Scale is completed by computing the mean scores for each of the three indicators before summing the indicator scores.

Subjects receive 0 points for answering "YES" and 1 point for answering "NO" to each of the activity-specific questions. [see Figure 1] Mean scores are computed for each of the three indicators: planning for participation, interference with participation, and prevention from participation. Indicator scores have potentials to range from 0–1 with higher scores reflective of less planning, less interference, and less prevention or rather increased participation. Since each indicator score is the mean across five activities, the variables are considered approximately continuous. Computing the sum across all three indicators completes scoring. Total scores have potentials to range from 0–3.

#### Content validity

Face and content validity were addressed through the manner in which items were generated from statements and themes from qualitative interviews and through expert review. Face validity was evaluated by four adolescents with asthma, three parents of school-age children with asthma, and a representative of the American Lung Association. Content validity was evaluated by two physicians, two advance practice nurses, and a respiratory therapist specializing in asthma or pediatric pulmonary medicine; a psychologist and a social worker who counsel children with asthma; and three researchers experienced in instrumentation. A standardized form was used to evaluate the scale.

Reviewers were in agreement that the instrument appeared sound and relevant with a logical tie between the purpose and items. Directions were deemed clear, logical, appropriate, and free of excess wording. Questions were considered grammatically correct, clear in meaning, conveying a single thought, appropriate for the response choice, and free of excess wording. Choice options were judged to be clearly defined, appropriate for the instrument and target population, arranged in a logical order, and grammatically correct. Content was deemed relevant and consistent with theoretical expectations without areas of omission.

### Testing of the PLA

#### Design

A cross-sectional design was used. The study was in full compliance with the Helsinki Declaration and Health Insurance Portability and Accountability Act (HIPAA) requirements. Data from three studies were combined to evaluate completion rates. Prior to data collection, human subjects' approvals were obtained through the University of Arizona Health Sciences Center Review Board for subjects recruited primarily in Arizona (1995–1996), the University of Michigan Health Sciences Institutional Review Board for subjects recruited in Michigan and Ohio (2001–2004), and Michigan State University Biomedical Institutional Review Board for subjects recruited in south central Michigan (2005–2007). For all studies, written consent was obtained from a parent or legal guardian and assent from the child.

#### Sample and setting

The convenience sample consisted of 313 children, ages 9–15 years (*M *= 11.53, *SD *= 1.62), who lived in northern lower, south-eastern and south-central Michigan (*n *= 14, 4.5%, *n *= 35, 11.1%, and *n *= 153, 48.9%), southern Arizona (*n *= 80, 25.6%), north-western Ohio (*n *= 27, 8.6%), and central Oklahoma (*n *= 4, 1.3%).

#### Return rates

For the first two studies, of the 318 paper-and-pencil packets mailed, 219 (69%) were returned. For the third study, of the 109 families approached, 94 (86%) were recruited, enrolled, kept appointments for data collection, and completed the surveys. Demographic data are presented in Tables 2, 3 and 4.

**Table 2:** Cross-group Comparisons for PLA Scores between Males and Females

	Males (*n *= 157, 52%)		Females (*n *= 147, 48%)				
	*M*	*SD*	*M*	*SD*	*t*	*df*	*p*
Think About Participation	.486	.332	.478	.337	.185	302	.853
Interferes with Participation	.618	.306	.551	.333	1.834	302	.068
Prevention from Participation	.815	.295	.678	.317	3.906	302	.000*
Participation in Life Activities	1.919	.742	1.707	.817	2.365	302	.019*

*p-value significant < .05

**Table 3:** Cross-group Comparisons for PLA Scores between African American/Black and Non-Hispanic Caucasian American/White Participants

	Black (*n *= 69, 23%)		White (*n *= 177, 58%)				
	*M*	*SD*	*M*	*SD*	*t*	*df*	*P*
Think About Participation	.491	.362	.495	.334	-.074	244	.941
Interferes with Participation	.549	356	.636	.301	-1.975	244	.049
Prevention from Participation	.696	.366	.798	.277	-2.079	99†	.040*
Participation in Life Activities	1.737	.914	1. 929	.716	-1.569	102†	.120

*p-value significant < .05

†Levene's Test for Equality of Variances indicated equal variances were not assumed.

**Table 4:** Cross-group Comparisons in PLA Scores by Age, Race, Socioeconomic Status, and Area of Residence

Groupings	*N*	*M*	*SD*		*Sum of Squares*	*df*	*Mean Square*	*F*	*p*
Age									
9–10 years	87	1.839	.845	Between	3.196	4	.799	1.303	.27
11 years	75	1.684	.744						
12 years	55	1.791	.781	Within	183.425	299	.613		
13 years	52	1.845	.818						
14–15 years	35	2.040	.639	Total	186.622	303			
Total	304	1.816	.785						
									
Race ‡									
African American/Black	69	1.737	.914	Between	6.505	4	1.626	2.700	.03^ns^
Hispanic/Latino(a)	21	1.552	.869						
Caucasian/White	177	1.929	.716	Within	180.116	299	.602		
Mixed & Others	25	1.525	.782						
Missing	12	1.675	.555	Total	186.622	303			
Total	304	1.816	.785						
									
Socioeconomic Status									
lower 0–49 points	86	1.615*	.852	Between	8.720	3	2.907	4.941	.00*
low middle 50–69 points	84	1.825	.767						
upper middle 70–89 points	76	1.825	.744	Within	175.889	299	.588		
upper 90–99 points	57	2.119*	.652						
Total	303	1.821	.782	Total	184.609	302			
									
Residence									
So Arizona/California	84	1.812	.755	Between	1.166	4	.291	.470	.76
North western Ohio	27	1.859	.803						
Northern Lower Michigan	14	1.986	.523	Within	185.456	299	.620		
South eastern Michigan	34	1.924	.948						
South central Michigan	145	1.769	.782	Total	186.622	303			
Total	304	1.816	.785						

*p-value significant < .05

†Harmonic mean used due to unequal group sizes.

‡Others included Asian, Pacific Islander, Middle Eastern, Native American

#### Data collection

Data were collected from children diagnosed with asthma, ages 9–15 years, who were able to read and understand English. Flyers advertising the studies were offered to families through physicians' offices and schools. Families interested in learning about the studies contacted the PI. After being informed of the purpose and nature of the study, requirements and responsibilities of subjects, and risks and benefits, families agreeing to participate in the first two studies were mailed a questionnaire packet. For the third study, home visits were scheduled for data to be collected using laptop computers. All items were entered into a user-friendly data entry system. The system was audio-linked so that when participants clicked on icons, items and response options were read aloud in English.

The questionnaire packets contained a cover letter, legal guardian consent and child assent forms, two questionnaire booklets, and an envelope with return prepaid postage. The child completed one booklet and a parent/caregiver completed the other. One week after the packets were mailed, families were contacted by telephone and asked if they needed any assistance. For the third study, trained evaluators obtained consent and assent, and assisted as needed with completion of the surveys loaded on laptop/notebook computers. In addition to the PLA, children were asked to complete 5–7 additional instruments depending on the study. Parents were asked to complete the General Health History Survey (GHHS) and three additional instruments. The GHHS is described below.

#### Demographic data

The *General Health History Survey *is a 36-item survey completed by parents designed to collect demographic and disease-related information [13-15]. Demographic information reported here relates to age, sex or gender, race, residence by area of state, and socioeconomic status. Socioeconomic status was computed using the Nam-Powers Socioeconomic Index Scores (SEIS) by averaging parents' occupation and education scores, and family income score [27]. The SEIS has demonstrated an extremely high degree of stability in status scores with correlation coefficients of .97 over 10 years, and .91 over 20 years [28].

#### Monetary Award

Families that returned completed questionnaires were offered an award of $5 for the first study, $10 for the second study, and $30 for the third study. For the first two studies, healthcare providers who recruited eligible subjects were paid $5 per family that returned completed questionnaires. For the third study, school nurses were reimbursed for the time they served as recruiters on the study.

#### Data analysis

SPSS for Windows 14.0.2 [29] was used to recode and score the instruments. Descriptive statistics were used for the General Health History Survey. The Socioeconomic Index Score was computed by averaging three composite scores. Independent samples t-tests and analysis of variance were used for cross-group comparisons.

#### Power analysis

This study was part of a series of studies designed to evaluate psychometric properties of newly developed instruments. In determining sample size, the number of items contained in the target instruments, sensitivity of other instruments being used, and data analysis techniques were considered. Based on equations provided by Kim [30], for evaluating psychometric properties using confirmatory factor models for larger instruments contained in the packet, sample size required a minimum of 214 participants.

## Results

### Completion rate

This survey was presented as fourth in a series of questionnaires. Completion rate of all surveys including the PLA was 97%. Nine subjects chose to stop prior to this instrument. Those completing the PLA were able to identify their favorite activities and answer the three questions. Thirteen subjects identified three to four activities but left the others blank. Ten subjects entered two of their favorite activities in the space provided for one activity (i.e., reading and writing or football and basketball). One subject wrote "sports" on each line without specifying the type of sport.

Some subjects wrote comments clarifying or explaining their response choices. For example, one subject wrote that asthma interfered with reading when the books were dusty. Phonetic spelling of activities was interesting, although not difficult to decipher. Formal names and acronyms of specialized activities and youth groups were challenging when classifying activities. Knowledge of the population was important. For example, *folklorico *is a highly energetic form of Mexican folk dancing.

Subjects enjoyed the paper-and-pencil instrument. Most subjects circled ALL of their favorite activities before selecting five. Some drew pictures of themselves actively engaged in activities or despondently watching as others engaged in activities while they struggled with breathing difficulties. A printed handout listing activities was offered to subjects using the audio-linked data entry system to support their completion of the survey items.

### Scores

Actual scores for all three indicators ranged from 0–1 with higher scores reflective of less restriction or rather increased participation. The mean score of planning was .482 (*SD *= .334), interference was .586 (*SD *= .320), and prevention was .749 (*SD *= .313). Overall participation in life activities scores ranged from 0–3 (*M *= 1.816, *SD *= .785). Skewness of the overall score was -.556 and Kurtosis was -.279.

For this cross-sectional sample of children responding to questions prior to delivery of any formal asthma health education or counselling interventions, all three indicator scores functioned as predicted. Mean scores indicated that for the combined sample approximately 52% of the time children considered their asthma when planning for favorite activities, 42% of the time asthma interfered with favorite activities, and 25% of the time asthma prevented participation in favorite activities.

### Cross-group comparisons

Cross-group comparisons of the three indicator mean scores and overall participation summed scores are presented in Tables 2, 3 and 4. Although preliminary tests revealed no significant differences in overall participation scores based on age, race or residence groupings, significant difference were indicated between males (*M *= 1.92, *SD *= .74) and females (*M *= 1.71, *SD *= .82), *t*(302) = 2.365, *p *= .02, as well as the highest (*M *= 2.12, *SD *= .65) and lowest (*M *= 1.62, *SD *= .85) socioeconomic groups (*p *= .002).

In addition, the prevention from participation mean score for males (*M *= .82, *SD *= .30) was significantly higher than females (*M *= .68, *SD *= .32) indicating that females were prevented from participation by their condition more often than males, *t*(302) = 3.906, *p *= .001. Prevention from participation mean scores were also significantly different based on race between black (*M *= .70, *SD *= .37) and white (*M *= .80, *SD *= .28) subjects, *t*(99) = -2.079, *p *= .04, indicating that black subjects were prevented from participation by their condition more often than white subjects.

When accounting for unequal group sizes, post-hoc analysis revealed no significant difference in overall participation scores based on race. Clearly, more research is needed with diverse populations, specifically targeting Hispanic/Latino, Pacific Islander, Middle Eastern, and Native American groups.

## Discussion

This paper described development of the PLA and reported on face and content validity of the instrument designed to measure one aspect of quality of life defined as level of unrestricted involvement in chosen pursuits. Unique contributions to scale development and implications of the instrument for theory development, future research, and clinical practice are discussed below.

### Scale Development

The concept of focus for development of this scale was identified and defined through themes extracted from qualitative interviews with adolescents identified as accepting of their asthma. Indicators for the concept evolved from participants' statements. Level of participation in activities was isolated as the prime motivator for behavioral changes in coming to accept asthma as a chronic condition requiring ongoing monitoring and management [12]. Although a few global quality of life instruments [22-25] contain items that address physical activities and limitations, based on theoretical and empirical findings, the PLA provides an extension of the typical biological, psychological, social and spiritual quality of life dimensions in existence. Focusing on dimensions of participation in life activities in concert with asthma remissions and exacerbations is a strength of the PLA.

By having participants select their own activities, responses to the PLA are individualized in meaningful ways not offered by the more global subscales of the PedsQL™ [25] or AAQOL [23]. Providing an extensive list of fun things to do including a broad range of recreational opportunities, memberships in organized clubs or youth groups, options for individual craft or art projects, and choices of both indoor and outdoor sport alternatives prompts identification and selection of one's most favorite activities.

Unique to this instrument is the idea that the activity or classification of activity referred to by the questions is not as important as whether or not planning is required and/or participation is disrupted or limited. The PAQLQ [24] asks children to identify activities that were limited due to their asthma in the recent past, important to the child, and performed frequently, but does not allow the behavior to change over time. Allowing activities to change in interest and vary in number with seasons and over time offers children opportunities to grow and develop through adolescence into adulthood by ever increasing in complexity, differentiation, and specialization, as well as hierarchical integration and organization.

Indicators measuring levels of planning for participation, interference with and prevention from participation afford dimensions of the concept that distinguish the PLA from other scales. The PedsQL™ [25] measures level of difficulty specifically related to two activities without clearly defining what is meant by how hard. The question must be asked, What about engaging in the activities is hard? The AAQOL [23] measures how frequently symptoms happen and the importance of symptoms associated with specific events without addressing whether or not activities are limited, restricted or prevented.

### Face and Content Validity

Results of this study determined face and content validity of the PLA to be acceptable and relevant, respectively. Completion rate across all three studies was high. Students as young as grade 3, age 9 years, were able to complete the instrument. From a lifespan development perspective the instrument was deemed suitable for students enrolled in grades 3–11.

Once face and content validity are established, testing for purposes of estimating internal consistency reliability and construct validity of the instrument can be explored. Unlike the LAQ [22]and PAQLQ [24], the structure and format of the PLA lend well to psychometric testing, specifically internal consistency reliability and construct validity using factor analysis techniques. If the instrument demonstrates sound psychometric properties of internal consistence reliability, stability, and construct validity, the PLA could be used for theory testing and to evaluate the efficacy and effective of treatments and interventions designed to foster increase participation in life activities. Implications of the PLA for use in theory testing, research settings, and clinical practice are discussed below.

#### Theoretical implications

The concept of participation in life activities as a measure for child and adolescent quality of life possesses implications for theory development. Findings of this study provide preliminary support for the qualitatively-derived theoretical underpinnings of the instrument. The PLA contributes to the advancement of science by offering a tool to measure what is hypothesized to be the primary motivator for child and adolescent behavioral change and psychosocial acceptance of the chronic condition [12,15].

In preparation for theory testing, relationships between participation in life activities and social, psychological, and biological well-being should be considered. Evidence suggests that for this target age group, support from healthcare professionals, parents, caregivers, and best friends fosters participation in life activities [13,14], and consequently, participation in life activities enriches psychosocial outcomes such as self-perception of athletic competence, physical appearance, social acceptance, and global self-worth, as well as perceived social support from classmates and schoolteachers [13,14]. The impact of increased participation in life activities on biological or physical outcomes could be tested using the PLA.

#### Research implications

With adequate sample size and completion rates, the logical next step is to evaluate psychometric properties of internal consistency reliability and construct validity. In addition to factor analysis, predictive concurrent techniques to explore hypothesized associations with related concepts (i.e., school days missed), convergent instruments (i.e., quality of life measures), and contrasting groups (i.e., children with asthma ranging from mild intermittent to severe persistent conditions, children without asthma or children with conditions other than asthma) would provide valuable information. Convergent validity of the PLA could certainly be evaluated using the AAQOL physical activity subscale [23]. Effect size and clinical appropriateness will also need to be established. Longitudinal methods will be needed to evaluate abilities to capture stability and change over time.

When examining internal consistency reliability and construct validity of the PLA, sex/gender, race, and socioeconomic status will need to be considered. Preliminary cross-group comparisons indicated significant difference in PLA scores between males and females, and lowest to highest socioeconomic groups. More research is needed to explore similarities and differences in scores based on race between Black and White Americans. Comparing and contrasting activities selected by males and females is worth of pursuing, specifically related to the potential for exposure to stimuli that might exacerbate symptoms. Comparing and contrasting severity of illness ratings and asthma management plans based on sex/gender, race, and socioeconomic groups is of particular interest.

#### Clinical implications

With face and content validity established, the PLA is ready for testing in clinical settings. In clinical settings the PLA could be used to lead discussions designed to motivate behavioral change in child and adolescent management of asthma. Having children as young as age 9 years complete the PLA during interactions with their healthcare providers could offer entry into discussions to provide the foundation for goal setting. Assessing levels of planning, interference, and restriction related to participation in specific activities could offer opportunities for information processing related to reasoning about management of acute episodes of symptom exacerbation as well as problem-solving and decision-making related lifelong condition management. Asthma action plans could be tailored to increase participation in self-selected favorite activities. Over time, the PLA could be used to evaluate the efficacy and effectiveness of treatments and interventions designed to improve quality of life.

## Conclusion

Face and content validity of the PLA was determined to be highly acceptable and relevant by lay and expert reviewers. The qualitatively-derived and theoretically-based instrument was deemed appropriate, useful, and applicable for both males and females ranging in age from 9–15 years of African American and Caucasian American origins and from varying socioeconomic backgrounds.

## List of abbreviations

PLA: Participation in Life Activities Scale; LAQ: Life Activities Questionnaire for Childhood Asthma; PAQLQ: Pediatric Asthma Quality of Life Questionnaire; ICC: Interclass Correlation; PedsQL™: Pediatric Quality of Life Inventory™ Generic Core Scales and Asthma Module; AAQOL: Adolescent Asthma Quality of Life Questionnaire; HIPAA: Health Insurance Portability and Accountability Act; SEIS: Nam-Powers Socioeconomic Index Scores

## Competing interests

The author declares that they have no competing interests.

## Authors' contributions

The author is solely responsible for the content contained in this article.

## Supplementary Material

Additional file 1 **Participation in Life Activities Scale**. The form completed by children and adolescents diagnosed with asthma is titled, "*My Favorite Things to Do*." Click here for file
