# Supplementary material for: Development and preliminary evaluation of the participation in life activities scale for children and adolescents with asthma: an instrument development study
Source: Health Qual Life Outcomes. 2008 May 28;6:37. doi: 10.1186/1477-7525-6-37 (PMC2429900; doi:10.1186/1477-7525-6-37)
Supplement: Additional file 1 — Participation in Life Activities Scale. The form completed by children and adolescents diagnosed with asthma is titled, "My Favorite Things to Do." [file 1477-7525-6-37-S1.pdf]

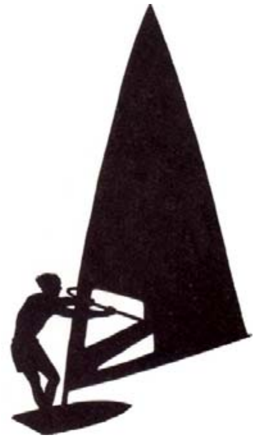

My Favorite Things To Do

This will be fun!!

I want to know if your asthma ever keeps you from doing things that you want to do. I want you to think about your most favorite things to do and tell me if your asthma ever keeps you from doing them.

Directions:

Write your five most favorite activities on the lines provided on page 4. After listing your favorite activities, read each of the questions below the line and answer them by circling either "Yes" or "NO".

NOTE: I have given you a list on page 2 of some things people like to do to get you started thinking. You may choose from the list or <u>if your most favorite thing is not on the list you can add it</u>.

The examples on page 3 may help guide your thinking in answering the questions.

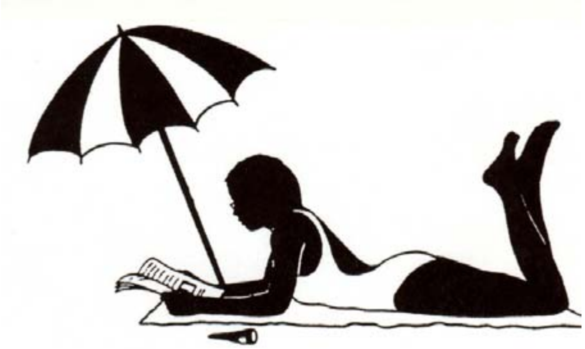

© Copyright Eileen Kae Kintner 1996

List of Fun Things To Do

Amusement parks
Art Work
Backpacking
Camping
Clubs
Cooking
Crafts
Dancing
Gardening
Going to Concerts

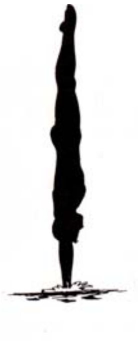

Going to the Library
Going to the Mall
Going to the Movies
Photography
Playing Cards
Playing Outside
Playing Inside
Reading Books
Singing
Playing a Musical Instrument

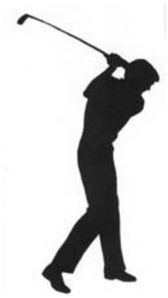

Stamp Collecting
Taking care of Pets
Talking on the Phone
Traveling
Visiting Art Galleries
Visiting Museums
Visiting with Friends
Watching Sports
Watching TV
Writing

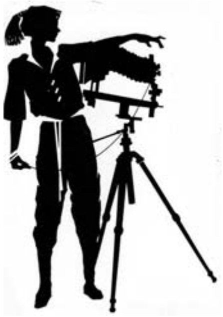

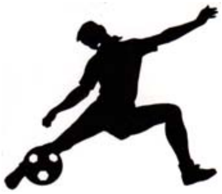

Clubs - Youth Groups

4-H, Scouts
Band or Choir
Drama

Student Council
School-related Groups
Other than School-related Youth Groups

Crafts-Arts

Art work
Building Models
Boats, Airplanes, Space Ships
Crocheting, Knitting, Macramé

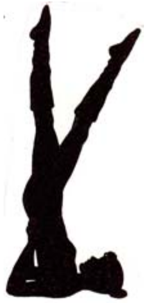

Painting
Pottery
Sewing
Woodwork

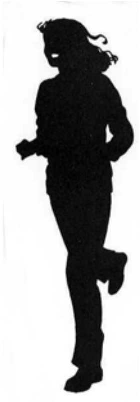

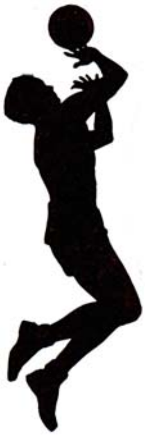

Sports – Indoor or Outdoor

Archery	Fishing	Hunting	Snowshoeing
Baseball	Golf	Pool	Soccer
Basketball	Gymnastics	Racquetball	Swimming
Biking	Hiking	Scuba Diving	Tennis
Bowling	Hockey	Skating	Volleyball
Cheerleading	Horseback Riding	Snow Skiing	Water Skiing

© Copyright Eileen Kae Kintner 1996

My Favorite Things To Do

The following examples might help you in answering the questions.

Do you need to think about your asthma when planning to do this activity?

"YES" Example:

John plans to go running this afternoon. He needs to think about his asthma before running. He has learned from experience that he will need to use his inhaler before running in order to keep from having breathing problems.

"NO" Example:

John is planning to go to the library this afternoon to study. He does <u>not</u> need to think about his asthma before going to the newly built air-conditioned library.

Does your asthma interfere with your participating in this activity?

"YES" Example:

Lisa joined a group of friends on a late afternoon horse-drawn hayride and evening bonfire. While at the bonfire, Lisa had some problem breathing. She used her inhaler and after sitting quietly for about 10 minutes was able to rejoin the group.

"NO" Example:

Lisa had some friends over to her house to bake a cake. She visited with her friends all afternoon and never had any breathing problem.

Does your asthma keep you from participating in this activity?

"YES" Example:

Chris likes to swim very much. He wants very much to go deep-sea scuba diving. The scuba diving instructor and his doctor both said scuba diving is dangerous and sometimes life threatening for some people with asthma.

"NO" Example:

Chris likes to swim very much. At the seashore, she enjoys snorkeling and can swim around for hours without any problems.

© Copyright Eileen Kae Kintner 1996

My Favorite Things To Do

Directions: List your five most favorite activities in the blank lines. After listing your favorite activities read each of the questions below and answer them by circling either "Yes" or "NO".

Favorite Activity #1____

Do you need to think about your asthma when planning to do this activity?	YES	NO
Does your asthma interfere with your participating in this activity?	YES	NO
Does your asthma ever keep you from participating in this activity?	YES	NO

Favorite Activity #2____

Do you need to think about your asthma when planning to do this activity?	YES	NO
Does your asthma interfere with your participating in this activity?	YES	NO
Does your asthma ever keep you from participating in this activity?	YES	NO

Favorite Activity #3____

Do you need to think about your asthma when planning to do this activity?	YES	NO
Does your asthma interfere with your participating in this activity?	YES	NO
Does your asthma ever keep you from participating in this activity?	YES	NO

Favorite Activity #4____

Do you need to think about your asthma when planning to do this activity?	YES	NO
Does your asthma interfere with your participating in this activity?	YES	NO
Does your asthma ever keep you from participating in this activity?	YES	NO

Favorite Activity #5____

Do you need to think about your asthma when planning to do this activity?	YES	NO
Does your asthma interfere with your participating in this activity?	YES	NO
Does your asthma ever keep you from participating in this activity?	YES	NO

© Copyright Eileen Kae Kintner 1996
